# Change in healthcare professionals’ perception of self-efficacy for providing dietary advice after completing a massive open online course: an uncontrolled community trial in 64 Brazilian municipalities, 2022-2024

**DOI:** 10.1590/S2237-96222025v34e20240330.en

**Published:** 2025-06-13

**Authors:** Vanessa Del Castillo Silva Couto, Adhara Brandão Lima Vanhoz, Kamila Tiemann Gabe, Cleyton Zanardo de Oliveira, Patrícia Constante Jaime

**Affiliations:** 1 Universidade de São Paulo, Núcleo de Pesquisas Epidemiológicas em Nutrição e Saúde, São Paulo, SP, Brazil; 2 Escritório do Programa de Apoio ao Desenvolvimento Institucional do Sistema Único de Saúde, BP – A Beneficência Portuguesa de São Paulo, São Paulo, SP, Brasil

**Keywords:** Dietary Guidelines, Nutritional Policy, Professional Education in Public Health, Remote Education, Evaluation Study, Guías Alimentarias, Política Nutricional, Educación Profesional en Salud Pública, Educación a Distancia, Estudio de Evaluación

## Abstract

**Objective:**

To assess the change in the perception of self-efficacy of Primary Health Care professionals to provide dietary advice using the Protocols based on the Brazilian Dietary Guidelines for Individual Dietary Advice, after completing a massive online open course (MOOC).

**Methods:**

Uncontrolled community trial. The study population comprises degree-holding healthcare professionals working in Primary Health Care in 64 small municipalities in Brazil. The intervention was a massive online open course. The outcome was assessed by a validated scale that measures the perception of self-efficacy to provide dietary advice based on the Protocols based on the Brazilian Dietary Guidelines. The mean score was calculated before and after the intervention and the change in score was tested with paired t-test (p-value<0.05).

**Results:**

Among the 1,201 health professionals, the average self-efficacy score increased from 23.71 to 37.31 after the intervention (p-value<0.001), an increase that was significant in all professional categories.

**Conclusion:**

The massive online open course increased the perception of self-efficacy of Primary Health Care professionals to provide individual dietary advice based on the Protocols based on the Brazilian Dietary Guidelines.

## Resumen

**Objetivo:**

Evaluar el cambio en la percepción de la autoeficacia de los profesionales de la salud de atención primaria para proporcionar orientación alimentaria utilizando los Protocolos de Utilización de la Guía Alimentaria para la Población Brasileña después de un curso en línea masivo y abierto (MOOC, por su sigla en inglés).

**Métodos:**

Ensayo comunitario no controlado. La población del estudio está conformada por profesionales de la salud de nivel superior de la atención primaria de salud en 64 municipios pequeños de Brasil. La intervención fue un curso en línea masivo y abierto. El resultado se midió a través de una escala validada que mide la percepción de autoeficacia para proporcionar orientación alimentaria basada en los Protocolos de Utilización de la Guía Alimentaria para la Población Brasileña. La puntuación media se calculó antes y después de la intervención y el cambio de la puntuación se probó con la prueba t pareada (p-valor<0,05).

**Resultados:**

Entre los 1.201 profesionales de la salud, la puntuación media de autoeficacia pasó de 23,71 a 37,31 después de la intervención (p-valor<0,001), un aumento que fue significativo en todas las categorías profesionales.

**Conclusión:**

El curso en línea masivo y abierto aumentó la percepción de autoeficacia de los profesionales de la salud de atención primaria para realizar orientaciones alimentarias individuales basadas en los Protocolos de Utilización de la Guía Alimentaria para la Población Brasileña.

**Ethical aspects**

This research respected ethical principles, having obtained the following approval data:

Research Ethics Committee: Opinion number, Approval date, Certificate of Submission for Ethical Appraisal

Hospital Beneficência Portuguesa de São Paulo: 6.121.289, 29/8/2022, 60875322.0.1001.5483

Universidade de São Paulo: 5.719.038, 24/10/2022, 60875322.0.2001.5421

Informed Consent Form: Obtido de todos os participantes antes da coleta.

## Introduction

The Brazilian Dietary Guidelines (hereinafter referred to as the Dietary Guidelines) is the document that establishes recommendations for healthy eating in Brazil, (1) based on evidence that supports its importance and relevance (2). Pioneering the incorporation of the Nova classification (3), which considers the level and purpose of food processing, the Dietary Guidelines also address dietary patterns, commensality, food culture and lists strategies to overcome barriers to healthy eating (4). The Dietary Guidelines have been implemented in the Brazilian Unified Health System (SUS) and in Primary Health Care (PHC) (5), serving as a reference for the development of other support tools for nutritional care and clinical practice, such as the Protocols based on the Brazilian Dietary Guidelines for Individual Dietary Advice (hereinafter referred to as Protocols) (6-8). Consisting of five fascicles organized by phases of the life course (9-13), the Protocols support health professionals in individual dietary advice, through a systematized process in three steps: food consumption assessment (14,15), organization of priority recommendations based on stepwise flowchart for decision-making, and implementation of specific dietary advice (6). 

Professional qualification is a medium-term strategy for the implementation the Dietary Guidelines and is a responsibility provided for in the SUS (5), which must ensure the qualification of health professionals to offer the best care (16). The National Policy for Continuing Health Education recommends basing health training on identified health needs, using reflective activities that consider the challenges of the work process, involving different actors, with the aim of promoting transformation in care practices (17). 

Massive Open Online Courses (MOOC) are a type of training characterized by the broad dissemination of educational content, via the internet, to large groups of students distributed across extensive territories. MOOC have been widely used in healthcare training around the world (18) and in the context of the SUS, as a tool for health professionals qualification (19-21).

Based on this scenario, a MOOC on the Protocols was developed and validated with the aim of qualifying PHC professionals. This study aimed to evaluate the change in perception of self-efficacy of Primary Health Care professionals to provide dietary advice using and the Dietary Guidelines and the Protocols after completing the course.

## Methods

### Study design and context

This study was an uncontrolled community trial. Participants were degree-holding health professionals working in PHC in 64 small municipalities in Brazil. The intervention consisted of professional qualification through a MOOC, called QualiGuia. The course aimed at supporting the incorporation of Protocols into the clinical practice of PHC health professionals. A change in the perception of self-efficacy to carry out dietary advice using the Protocols, influenced by the course, was expected from the participants. The outcome of interest was measured before and after the intervention, using a validated questionnaire to measure the perception of self-efficacy. This study was developed between 2022 and 2023, as part of a project, also called QualiGuia, of the SUS Institutional Development Support Program (PROADI-SUS) conducted by Beneficência Portuguesa Hospital, in São Paulo(22). 

### Study participants

64 small municipalities (5 to 30 thousand inhabitants) were selected, 62 from the main list and 2 substitutes, which agreed to participate and to offer the MOOC to their health professionals. The selection was based on the following criteria: receiving federal funding for the development of Food and Nutrition actions, distribution in the five macro-regions of the country, representation of municipalities with and without a nutritionist or dietitian (considered as synonyms) in the PHC and good performance in the coverage of nutritional status in the National Food and Nutrition Surveillance System. The MOOC was offered in each municipality, and 1,365 PHC health professionals started it. Of these, 1,269 completed the GAB2 questionnaire, Extended Part A before the training and 1,201 completed the course and completed the questionnaire again after the training. 

### Intervention design: QualiGuia MOOC

The MOOC was developed and validated within the scope of the PROADI-SUS QualiGuia project. This course was developed based on the need identified by the Ministry of Health to implement the Protocols, launched between 2021 and 2022, to support individual dietary advice by stages of life and advance the implementation of the Dietary Guidelines in PHC. 

This course, classified as a MOOC because it is online, accessible to a wide audience and covering a large territory, explores different pedagogical tools for distance education. The QualiGuia MOOC is available on the SUS Open University platform (23).

The course had as its theoretical framework a) The National Policy for Continuing Health Education, which guides the training based on needs identified in everyday life to promote reflections and changes in care (17); b) The National Primary Care Policy, reinforcing PHC as the locus for nutritional care qualification (24); and c) The National Food and Nutrition Policy, which defines guidelines for nutritional care and the promotion of adequate and healthy eating (25). 

The core content of the course is based on the Dietary Guidelines and the Protocols. Case studies based on the PHC context, presented as animated videos, are the main learning resource to stimulate reflection on ways of using the Protocols as a tool for nutritional care. 

The course was validated by a panel of 15 experts who evaluated clarity, relevance and adequacy of the theoretical framework (26). The written assessments were analyzed using the Content Validity Index (CVI). Additionally, two focus groups were analyzed using a comprehensive approach with thematic content analysis(26,27). The CVI was calculated by the proportion of Likert scale 3 and 4 scores, with all activities achieving CVI>0.8 (cut-off point), validating the course. The thematic analysis identified three categories: 1) Relevance of the proposal and recognition for the PHC: highlighting the strategic role of the MOOC in the implementation of the Dietary Guidelines. 2) Adequacy of content: reinforcing the quality of the material and teaching-learning methodology. 3) Challenges for the course implementation in PHC: pointing out relevant reflections for implementation. The format and structure of the course remained unchanged and the suggestions from the panel of experts were considered and incorporated into the final version.

The course is self-instructional, has a 30-hour workload and is aimed at degree-holding health professionals who provide one-to-one care. 

In this study, participants had one month to register and gain initial access to it, five months to complete the training, and an additional month to issue the certificate and save the materials.

### Source and data collection

Data was collected between January and November 2023 using the *Research Electronic Data Capture* - REDCap (28). Professionals were invited to complete the GAB 2 scale – Extended Part A online, immediately before the start and after the completion of the MOOC. Sociodemographic information and on prior knowledge about the Dietary Guidelines and Protocols were also collected before the course. 

### Outcome of interest and study variables

The perception of self-efficacy reflects personal confidence or competence in a certain ability to perform a specific behavior (29). Self-efficacy can indicate how people will act, helping to determine what they can do with the skills and knowledge acquired in the future (30). Furthermore, belief in self-efficacy influences choices and decisions, since people tend to prefer to carry out activities in which they feel confident and competent. 

The “self-efficacy measuring scale for application of the Dietary Guidelines by primary care health professionals” (named GAB 2 – part A) is a self-administered scale of 12 items in a four-point Likert scale (score from 0 to 3), allowing for scores from 0 to 36 points (31). Four additional items were developed to capture the following dimensions: 1) dietary assessment according to food consumption markers; 2) ordering of priority recommendations according to the stepwise flowchart for decision-making; 3) differentiation of specific dietary advice by stage of life; 4) a summary item of strategies for overcoming obstacles, with the golden rule of the Dietary Guidelines. 

The additional items were developed and discussed with researchers with experience in developing scales, the Dietary Guidelines and Protocols. After review, a second version of the items was evaluated by a PHC nutritionist to verify the extent of understanding of the items. 

The items were defined and the scale expanded to 16 items in a four-point Likert scale (score from 0 to 3), totaling from 0 to 48 points. Named GAB 2 – Expanded Part A, its internal consistency was assessed by Cronbach’s Alpha and a Factor Analysis was performed to verify the grouping of items. The evaluation used pre-intervention data from a subsample of 1,039 participants collected in July 2023. 

The understanding of the questions was satisfactory. The new scale presented a mean of 23.6; standard deviation of 10.7 and Cronbach’s alpha of 0.955 (95%CI 0.951; 0.959). The 12-item scale had a mean of 17.8; standard deviation of 8.6 and Cronbach’s alpha of 0.942 (95%CI 0.970; 0.976). The factor analysis had 2 components. Component 1: strategies for overcoming obstacles to healthy eating, brings together items related to confidence in supporting users to adopt a healthy diet according to the recommendations of the Dietary Guidelines. Component 2: individual dietary advice based on the Dietary Guidelines, brings together items that consolidate the knowledge of the recommendations of the Dietary Guidelines and practical applications to care according to the Dietary Guidelines and Protocols.

The scale adaptation was valid for measuring the outcome of interest, the perception of self-efficacy of PHC health professionals to provide individual dietary advice based on the Protocols and recommendations of the Dietary Guidelines (Supplementary Box 1).

Additionally, variables were produced with sociodemographic information (professional category, macro-region, date of birth, sex, race/skin color and years of experience in practice) and prior knowledge of the Dietary Guidelines and Protocols, with initial responses of “yes” and “no”. For those who answered “yes”, additional options were offered as: “only heard about it”, “already participated in some training” and “uses it in clinical practice”.

### Data analysis and measurement

Data were analyzed using Stata SE 16 and R Studio software (Version 4.3.2). The characterization of the participants was done with descriptive analysis of the qualitative variables described in frequency. To measure the change in the perception of self-efficacy, the total score (mean) of the GAB 2 scale – Expanded Part A was calculated for each individual, comparing the scores before and after taking the course with paired Student t-test. Associations between sociodemographic characteristics, prior knowledge about the Dietary Guidelines and Protocols and increased self-efficacy (considering the delta of the score variation) were analyzed using Student t-test and Anova. Tukey post-hoc analyses identified categories that showed significant association in the previous stage. Additionally, the characteristics of professionals who dropped out of the MOOC were compared with those who completed the course, applying Fisher’s Exact, Chi-Square and Student t-test to check the similarity between the groups. Statistical significance was accepted with p-value<0.050.

Supplementary Figure 1 shows a flowchart of the methodological steps of the study.

## Results

Among the 1,201 health professionals, the majority were female (79.3%), with 50.5% self-declaring as black and 47.5% as white. The most prevalent age group was 30 to 39 years of age (40.9%). The most represented professional category was Nursing (33.6%), followed by Dentistry (20.0%) and Medicine (15.2%). Around 49.1% had more than six years of experience in professional practice. The regions with the highest participation were Northeast (36.8%) and Southeast (31.7%) (Table 1). Furthermore, 53.0% of the professionals knew about the Dietary Guidelines, of which 51.3% had heard of it, while 22.4% used it in clinical practice. Regarding the Protocols, 41.2% stated that they knew them and of these, 48.0% had heard about them and 17.6% used them in clinical practice (Table 2). 

**Table 1:** Characteristics of healthcare professionals. Brazil, 2022-2024 (n=1,201)

Variables		n (%)
Professional categories		
	Nursing	404 (33.6)
	Dentistry	240 (20.0)
	Medicine	183 (15.2)
	Physiotherapy	100 (8.3)
	Nutrition	82 (6.8)
	Psychology	70 (5.8)
	Physical education	42 (3.5)
	Social Service	40 (3.3)
	Pharmacy, Speech Therapy and Occupational Therapy	40 (3.3)
**Macro-region of the country**		
	Northeast	442 (36.8)
	Southeast	381 (31.7)
	North	185 (15.4)
	South	155 (12.9)
	Midwest	38 (3.2)
**Race/skin color**		
	White	570 (47.5)
	Brown	517 (43.0)
	Black	89 (7.4)
	Yellow (Asian)	22 (1.8)
	Indigenous	3(0.2)
Sex		
	Female	953 (79.3)
	Male	248 (20.6)
**Age (years) (6 missing)**		
	≤29	350 (29.1)
	30-39	491 (40.9)
	40-49	248 (20.6)
	≥50	106 (8.8)
**Length of experience in the profession (years)**		
	≤1	203 (16.9)
	2-3	250 (20.8)
	4-5	158 (13.2)
	≥6	590 (49.1)

**Table 2:** Prior knowledge of the Dietary Guidelines and Protocols based on the Brazilian Dietary Guidelines by health professionals. Brazil, 2022-2024

Prior knowledge		n (%)
**Knowledge about the Dietary Guidelines** (n=1,201)		
	Knows about the Brazilian Dietary Guidelines	636 (53.0)
**Among those who know the Dietary Guidelines** (n=635)		
	Heard about it	326 (51.3)
	Participated in training	167 (26.3)
	Uses it in clinical practice	123 (19.4)
	Participated in training and uses it in clinical practice	19 (3.0)
**Knowledge about the Protocols based on the Brazilian Dietary Guidelines** (n=1,201)		
	Knows the Protocols based on the Diretary Guidelines	495 (41.2)
**Among those who know the Protocols based on the Dietary Guidelines** (n=494)		
	Heard about it	237 (48.0)
	Participated in training	170 (34.4)
	Uses it in clinical practice	78 (15.8)
	Participated in training and uses it in clinical practice	9 (1.8)

**Figure 1:**
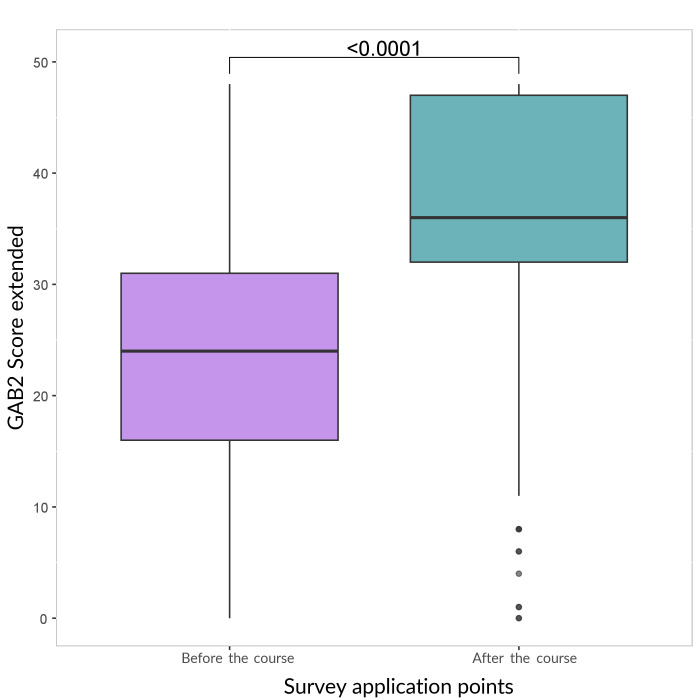
Distribution of the self-efficacy perception score to offer dietary advice based on the Protocols based on the Brazilian Dietary Guidelines before and after the intervention. Brazil, 2022-2024 (n=1,201)

Figure 1 shows the distribution of the professionals’ self-efficacy perception score after the intervention. Before the intervention, the mean self-efficacy score was 23.7 (median = 24; standard deviation = 10.7). After the intervention, the mean score increased to 37.3 (median = 36; standard deviation = 8.9), with a statistically significant difference (p-value<0.001). 

Table 3 shows the results by professional category, indicating a significant increase in the perception of self-efficacy in all professional categories after the intervention (p-value<0.001). Nutrition and Physical Education professionals showed the smallest percentage variations, while the other professional categories showed an increase of over 50%. 

**Table 3:** Difference in the self-efficacy perception score (mean, standard deviation, 95% CI) before and after the intervention, by professional category. Brazil, 2022–2024 (n=1.201)

		n	Pre-intervention self-efficacy score			Post-intervention self-efficacy score			Δ			p-value
Professional categories			Mean	Standard deviation	95%CI	Mean	Standard deviation	95%CI	Difference in means	Delta percentage	95%CI	
	Nursing	404	24.3	9.4	23.4; 25.2	37.6	8.2	36.8; 38.4	13.3	54.9	14.3; 12.3	<0.001
	Dentistry	240	20.0	10.4	18.7; 21.3	35.8	10.1	34.6; 37.1	15.8	79.2	17.3; 14.4	<0.001
	Medicine	183	24.8	8.9	23.5; 26.1	39.3	7.5	38.2; 40.4	14.4	58.1	15.8; 13.1	<0.001
	Physiotherapy	100	21.9	10.3	19.9; 24.0	37.9	8.8	36.1; 39.6	16.0	72.8	18.3; 13.7	<0.001
	Nutrition	82	38.2	8.6	36.3; 40.0	43.1	6.2	41.7; 44.5	4.9	12.9	6.3; 3.5	<0.001
	Psychology	70	19.2	10.9	16.6; 21.8	32.9	9.7	30.6; 35.3	13.7	71.3	16.8; 10.7	<0.001
	Physical education	42	25.9	8.8	23.1; 28.6	33.8	9.1	31.0; 36.7	7.9	30.7	10.8; 5.1	<0.001
	Social Service	40	21.3	11.9	17.5; 25.1	34.4	11.2	30.9; 38.0	13.1	61.4	16.5; 9.5	<0.001
	Pharmacy, Speech Therapy and Occupational Therapy	40	17.8	10.0	14.6; 21.1	34.8	6.9	32.6; 37.0	16.9	94.8	20.4; 13.4	<0.001

Table 4 details the association between sociodemographic characteristics, prior knowledge about the Dietary Guidelines and Protocols and increased self-efficacy. Among the sociodemographic characteristics, race/skin color, profession and macro-region were significantly associated with increased self-efficacy (p-value<0.050). The analysis revealed that the association was greater among white and black individuals. Nutrition and Physical Education professionals showed a smaller increase in self-efficacy compared to other professional categories such as Nursing, Dentistry, Medicine, Physiotherapy, Social Work, Psychology and Pharmacy, Speech Therapy and Occupational Therapy. For the macro-region variable, the association was greater between the South and Southeast macro-regions. 

**Table 4:** Association between sociodemographic characteristics and prior knowledge about the Dietary Guidelines and Protocols based on the Brazilian Dietary Guidelines with increased self-efficacy after the course (delta of score variation). Brazil, 2022-2024

Variables		n	Mean	Standard deviation	p-value
**Professional category**					<0.001
	Nursing	404	13.3	10.1	
	Dentistry	240	15.8	11.5	
	Medicine	183	14.4	9.2	
	Physiotherapy	100	16.0	11.6	
	Nutrition	82	4.9	6.5	
	Psychology	70	13.7	12.8	
	Physical Education	42	8.0	9.1	
	Social Service	40	13.1	11.1	
	Pharmacy, Speech therapy and occupational therapy	40	16.9	10.9	
**Macro-region of the country**					0.043
	Northeast	442	13.5	10.8	
	Southeast	381	12.9	10.9	
	North	185	13.3	10.6	
	South	155	16.0	10.6	
	Midwest	38	12.4	9.3	
**Race/skin color**					0.006
	White	570	14.6	10.8	
	Brown	517	13.0	10.7	
	Black	89	11.3	10.7	
	Yellow (Asian)/Indigenous	25	11.2	9.3	
Sex					0.261
	Female	953	13.8	10.9	
	Male	248	12.9	10.1	
**Age (years) (6 missing)**					0.149
	≤29	350	14.3	11.58	
	30-39	491	13.51	10.18	
	40-49	248	13.24	10.42	
	≥50	106	11.64	10.05	
**Length of experience in the profession** (years)					0.13
	≤1	203	13.0	9.8	
	2-3	250	13.8	11.1	
	4-5	158	13.1	11.6	
	≥6	590	13.8	10.6	
**Prior knowledge of the Dietary Guidelines**					
	Knows the Dietary Guidelines	636	11.7	10.1	<0.001
	Among those who know the Brazilian Dietary Guidelines:				
	Heard about it	326	13.1	9.8	0.336
	Participated in training	186	11.8	10.6	0.011
	Uses it in clinical practice	142	7.9	8.7	<0.001
**Knowledge about the Protocols based on the Dietary Guidelines**					
	Knows the Protocols based on the Dietary Guidelines	495	11.1	10.1	<0.001
	Among those who know the Protocols based on the Dietary Guidelines				
	Heard about it	237	12.4	10.3	0.061
	Participated in training	179	11.2	10.0	0.001
	Uses it in clinical practice	87	7.1	8.7	<0.001
					

Prior knowledge about the Dietary Guidelines and Protocols was also associated with increased self-efficacy (p-value<0.050). In both cases, the increase was significant among those who had already participated in training or who used it in their clinical practice (Table 4). 

Finally, when comparing the groups that completed and did not complete the course, significant differences were observed in relation to macro-region and gender. However, both groups were statistically similar in terms of prior knowledge about the Dietary Guidelines and Protocols (Supplementary Table 1). Furthermore, the mean self-efficacy scores before the intervention also did not show significant differences between the groups (Supplementary Table 2).

## Discussion

The results of this study corroborate the production of evidence on the role of professional qualification in increasing self-efficacy to offer dietary advice based on the Protocols and the Dietary Guidelines. After the intervention, a significant increase in the perception of self-efficacy was observed both for all professionals and for each professional category analyzed. 

The main limitation of this study is the absence of a control group, which restricts the ability to attribute the observed changes in self-efficacy exclusively to the training with the MOOC, as the results may have been influenced by unmeasured external factors. Another possible limitation is social desirability bias, since participants may have responded to the self-efficacy scale more positively upon completing the MOOC, believing that this would be the expected behavior, which does not always reflect the real perception of self-efficacy. However, it is important to highlight that self-efficacy can only be measured through self-administered scales. On the other hand, the study had strengths, such as its broad capillarity, with significant participation of professionals from all macro-regions and in a context similar to the reality of training carried out in this modality. Thus, the study itself serves as evidence of the effectiveness of this MOOC. 

Self-efficacy is a construct that can contribute to the evaluation of implementation, since it measures confidence in applying acquired knowledge in professional practice (31). Previous studies have indicated that there is a greater relationship between high self-efficacy and changes in practices than between high knowledge and changes in practices (32). Therefore, the significant increase in self-efficacy to offer dietary advice after training with the MOOC points to its potential to support changes in the care practices of health professionals, contributing to the implementation of the Protocols and the Dietary Guidelines in PHC.

The MOOC favored the increase in self-efficacy in all professional categories. Most professional categories showed a relative increase of more than 50% in the perception of self-efficacy after the intervention. The differences in self-efficacy means between professional categories decreased after the intervention, signaling a leveling between them. 

The Nutrition category presented the highest scores at both times, but the smallest relative increase in self-efficacy. This category centralizes the greatest knowledge in Food and Nutrition, but still benefited from the training, probably due to the characteristic of the Protocols to support the systematization of one-to-one care in clinical practice (33). 

These findings help to understand that the Dietary Guidelines and Protocols are not restricted to the core area of Nutrition, but address food in the field of collective health, with an emphasis on the purpose and processing of food, dietary patterns, eating habits, and food environment. In addition to bringing a new paradigm to food and nutrition, they address the main recommendation in the golden rule and have a series of strategies to overcome obstacles to healthy eating (34). These documents contribute to blurring the boundaries between specific knowledge of Nutrition and the interdisciplinary knowledge that makes up the field of public health. The MOOC acted as a mediator of this knowledge, promoting gains in trust both between specific categories and across interprofessional practices. 

Food and nutrition are recurring themes in PHC, given their relationship with poor nutrition and chronic diseases. Therefore, these are topics frequently addressed in care settings by different health professionals. The increase in self-efficacy to offer dietary advice based on the Dietary Guidelines is a positive result that reflects the interest and commitment of professionals in improving their practices for nutritional care, in addition to signaling advances in the practice of interprofessional collaboration (35). This result reinforces the identified need for specific training for individual dietary advice, allowing professionals to feel more confident in carrying out this care practice.

Prior knowledge about the Dietary Guidelines and Protocols was associated with increased self-efficacy among those who had already participated in previous training and/or already used these materials in their clinical practice. On the other hand, there was no association between increased self-efficacy and just having heard about the documents. This finding reinforces the relationship between knowledge and self-efficacy. Even so, there is an understanding that this type of course is based on the idea that training occurs throughout the professional trajectory, through the interaction between prior knowledge, daily work and new knowledge necessary to qualify care practices (36). 

MOOCs can be strategic tools for training health professionals in the Brazilian Unified Health System (SUS) (18,37). This study indicates that the MOOC is capable of increasing the confidence of health professionals to provide dietary advice based on the Protocols, regardless of their level of prior knowledge, promoting a leveling of self-efficacy among professional categories. 

The analysis also revealed that professionals who did not complete the MOOC were statistically similar to those who completed it in terms of prior knowledge about the Dietary Guidelines, Protocols and self-efficacy scores before the intervention. However, significant differences were seen regarding the macro-region, which reinforces regional disparities in the implementation of PHC in Brazil (38). This finding highlights the challenge of considering local contexts when planning health training actions.

Despite the advances, the challenge remains of planning and implementing professional qualifications in the SUS, ensuring that the best care practices are offered to the population (17). Among the challenges of MOOC-based professional training, we highlight the need to ensure that such training is reflective and connected to daily work, in addition to aiming to promote changes in care practices in health services(39). 

Future studies should evaluate the impact of this intervention at different levels, considering everything from professionals’ reactions to the MOOC to changes in behavior, indicators and other effects on the local health system. This will make possible to better understand the multiple dimensions that make up the impact assessment of this MOOC, taking into account the challenges of PHC and the local context (40). 

In conclusion, the MOOC course contributed to increasing the perception of self-efficacy of health professionals in offering individual dietary advice based on the Protocols based on the Brazilian Dietary Guidelines. This finding reinforces the evidence that a MOOC offered by the SUS can qualify health professionals and ensure the provision of quality training in the health system.

## Data Availability

To protect the privacy of the study participants, the database used in the study is not publicly available. However, an anonymized database can be made available by the lead author upon request.
