# Supplementary material for: Change in healthcare professionals’ perception of self-efficacy for providing dietary advice after completing a massive open online course: an uncontrolled community trial in 64 Brazilian municipalities, 2022-2024
Source: Epidemiol Serv Saude. 2025 Jun 13;34:e20240330. doi: 10.1590/S2237-96222025v34e20240330.en (PMC12176444; doi:10.1590/S2237-96222025v34e20240330.en)
Supplement: Supplementary file 1 [file 2237-9622-ress-34-e20240330-suppl1-en.pdf]

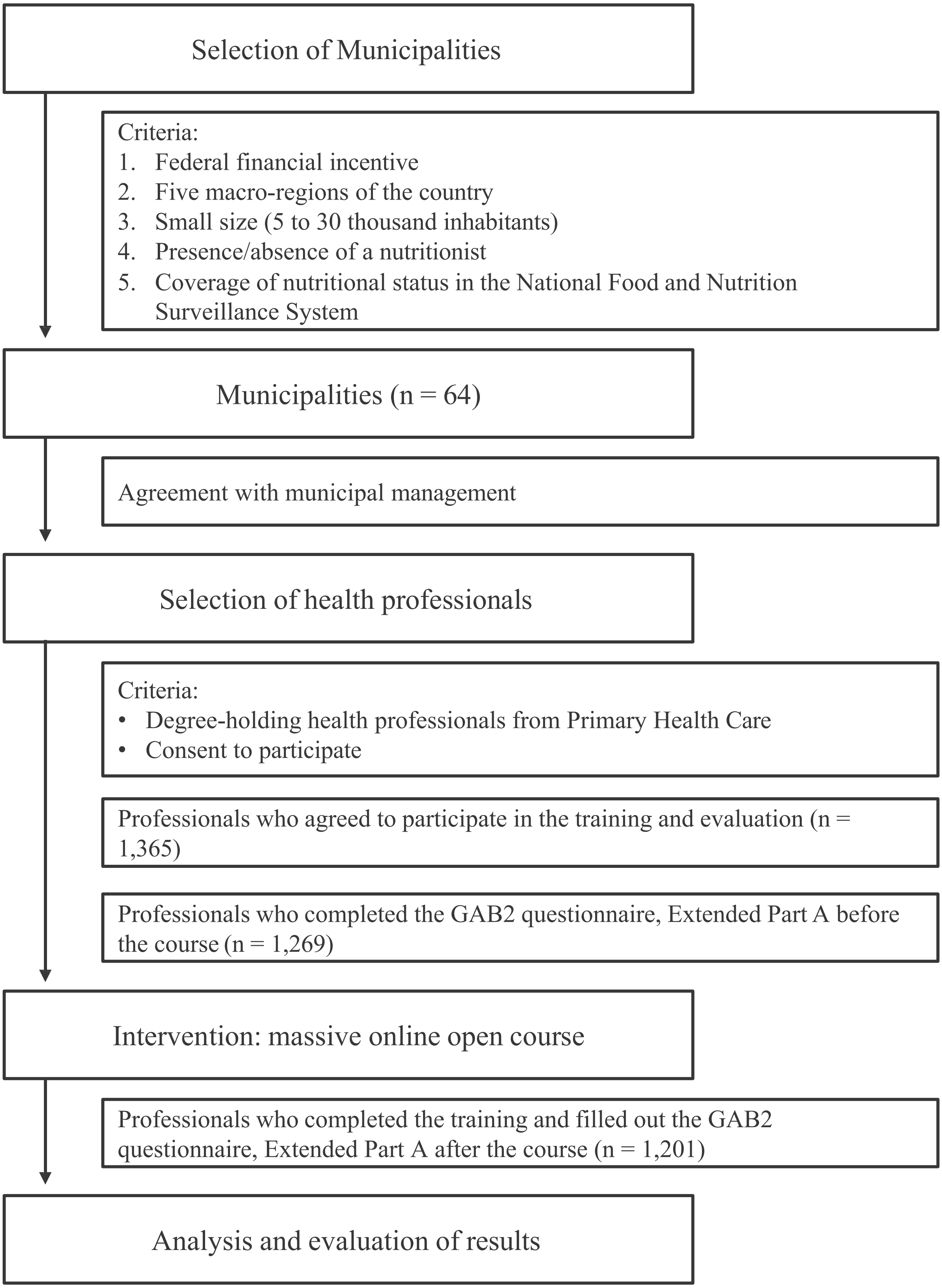
Selection of Municipalities
Criteria:
1. Federal financial incentive
2. Five macro-regions of the country
3. Small size (5 to 30 thousand inhabitants)
4. Presence/absence of a nutritionist
5. Coverage of nutritional status in the National Food and Nutrition Surveillance System
Municipalities (n = 64)
Agreement with municipal management
Selection of health professionals
Criteria:
• Degree-holding health professionals from Primary Health Care
• Consent to participate
Professionals who agreed to participate in the training and evaluation (n = 1,365)
Professionals who completed the GAB2 questionnaire, Extended Part A before the course (n = 1,269)
Intervention: massive online open course
Professionals who completed the training and filled out the GAB2 questionnaire, Extended Part A after the course (n = 1,201)
Analysis and evaluation of results

Supplementary Table No. 1 - GAB Scale 2 - Part A extended	
	Answers
In my daily work in Primary Health Care:	
1) I can advise healthcare service users on how to combine healthy foods into meals.	Not confident at all (0)<br>A little confident (1)<br>Confident (2)<br>Very confident (3)
2) I can teach healthcare service users how to analyze the ingredient list on food labels.	Not confident at all (0)<br>A little confident (1)<br>Confident (2)<br>Very confident (3)
3) I can encourage health service users to eat meals with their family or with other people whenever possible.	Not confident at all (0)<br>A little confident (1)<br>Confident (2)<br>Very confident (3)
4) I can promote the benefits of eating mindfully to healthcare service users.	Not confident at all (0)<br>A little confident (1)<br>Confident (2)<br>Very confident (3)
5) I can indicate reliable sources of information about healthy eating for healthcare service users.	Not confident at all (0)<br>A little confident (1)<br>Confident (2)<br>Very confident (3)
6) I can identify appropriate places within the community to purchase natural or minimally processed foods.	Not confident at all (0)<br>A little confident (1)<br>Confident (2)<br>Very confident (3)
7) I can help healthcare service users improve their cooking skills.	Not confident at all (0)<br>A little confident (1)<br>Confident (2)<br>Very confident (3)
8) I can stimulate the critical thinking among healthcare service users regarding food advertising.	Not confident at all (0)<br>A little confident (1)<br>Confident (2)<br>Very confident (3)
9) I can advise healthcare service users on how to save money when buying healthy foods.	Not confident at all (0)<br>A little confident (1)<br>Confident (2)<br>Very confident (3)
10) I can instruct healthcare service users on how to plan the use of time dedicated to eating.	Not confident at all (0)<br>A little confident (1)<br>Confident (2)<br>Very confident (3)
11) I know the contents of the Dietary Guidelines for the Brazilian Population, published by the Ministry of Health.	Not confident at all (0)<br>A little confident (1)<br>Confident (2)<br>Very confident (3)
12) I can promote healthy eating in my area of practice using the approach of the Dietary Guidelines for the Brazilian Population.	Not confident at all (0)<br>A little confident (1)<br>Confident (2)<br>Very confident (3)
During one-to-one care in Primary Health Care:	
13) I can assess the diet of healthcare service users according to SISVAN's (Food and Nutrition Surveillance System) food consumption markers.	Not confident at all (0)<br>A little confident (1)<br>Confident (2)<br>Very confident (3)

14) I can establish priorities in dietary advice for healthcare service users.	Not confident at all (0)<br>A little confident (1)<br>Confident (2)<br>Very confident (3)
15) I can provide specific dietary advice considering the life course of healthcare service users.	Not confident at all (0)<br>A little confident (1)<br>Confident (2)<br>Very confident (3)
16) I can suggest strategies for healthcare service users to reduce their consumption of ultra-processed foods and drinks.	Not confident at all (0)<br>A little confident (1)<br>Confident (2)<br>Very confident (3)

Supplementary Table No. 1 - Comparison between the characteristics and level of knowledge about the Dietary Guidelines and the Protocols based on the Brazilian Dietary Guidelines for Individual Dietary Advice of healthcare professionals who did not complete the training with those who completed the training (*n*=1,269), Brazil, 2022-2024

	Professionals who did not complete the course (*n*=68)	Professionals who completed the course (*n*=1,201)	*p*-value
	n (%)	*n* (%)	
Professional categories			
Nursing	26 (38.2)	404 (33.6)	
Dentistry	14 (20.6)	240 (20.0)	
Medicine	16 (23.5)	183 (15.2)	
Other professional categories	12 (17.6)	374 (31.1)	0.069
Regions of the country			
Northeast	29 (42.6)	442 (36.8)	
Southeast	16 (23.5)	381 (31.7)	
North	15 (22.0)	185 (15.4)	
South	3 (4.4)	155 (12.9)	
Midwest	5 (7.3)	38 (3.1)	0.021
Race/skin color			
White	35 (51.5)	570 (47.5)	
Brown	29 (42.6)	517 (43.0)	
Black	2 (1.1)	89 (7.4)	
Yellow (Asian)	2 (1.1)	22 (1.8)	
Indigenous	0 (0.0)	3 (0.2)	0.510
Sex			
Female	45 (66.2)	953 (79.3)	
Male	23 (33.8)	248 (20.6)	0.010
Age (years)			
≤ 29	16 (4.5)	337 (95.5)	
30-39	35 (6.7)	489 (93.3)	
40-49	15 (5.5)	259 (94.5)	
≥ 50	2 (1.1)	110 (98.2)	0.160
Length of experience in the profession (years)			
≤ 1	8 (11.8)	203 (16.9)	
2-3	21 (30.9)	250 (20.8)	
4-5	13 (19.1)	158 (13.2)	
≥ 6	26 (38.2)	590 (49.1)	0.062
Knowledge about the Dietary Guidelines			
Yes	38 (55.9)	636 (53.0)	
No	30 (44.1)	565 (47.0)	0.638

	Professionals who did not complete the course (n=68)	Professionals who completed the course (n=1,201)	p-value
	n (%)	n (%)	
Among those who know the Brazilian Dietary Guidelines:			
Heard about it:			
Yes	18 (26.5)	326 (27.1)	
No	50 (73.5)	875 (72.9)	0.903
Participated in training:			
Yes	11 (16,18)	186 (15.5)	
No	57 (83.8)	1,015 (84.5)	0.879
Uses it in clinical practice:			
Yes	10 (14.7)	142 (11.8)	
No	58 (85.3)	1,059 (88.2)	0.476
Knowledge about the Protocols based on the Brazilian Dietary Guidelines for Individual Dietary Advice:			
Yes	26 (38.2)	495 (41.2)	
No	42 (61.8)	706 (58.8)	0.627
Among those who know the Protocols based on the Brazilian Dietary Guidelines for Individual Dietary Advice:			
Heard about it:			
Yes	10 (14.7)	237 (19.7)	
No	58 (85.3)	964 (80.3)	0.308
Participated in training:			
Yes	9 (13.2)	179 (14.9)	
No	59 (86.8)	1,022 (85.1)	0.706
Uses it in clinical practice:			
Yes	8 (11.8)	87 (7.2)	
No	60 (88.2)	1,114 (92.8)	0.168

Supplementary Table No. 2 - Comparison between the perception of self-efficacy (mean, standard deviation, 95% CI) before the intervention of health professionals who did not complete the training with those who completed the training (*n*=1,269), Brazil, 2022-2024

	Professionals who did not complete the course (*n*=68)	Professionals who completed the course (*n*=1,201)	*p*-value
Pre-intervention self-efficacy score:			
Mean	23.1	23.7	
Standard deviation	10.7	10.7	
95% CI	20.5-25.7	23.1-24.3	0,671
