# Supplementary material for: Change in healthcare professionals’ perception of self-efficacy for providing dietary advice after completing a massive open online course: an uncontrolled community trial in 64 Brazilian municipalities, 2022-2024
Source: Epidemiol Serv Saude. 2025 Jun 13;34:e20240330. doi: 10.1590/S2237-96222025v34e20240330.en (PMC12176444; doi:10.1590/S2237-96222025v34e20240330.en)
Supplement: Supplementary file 2 [file 2237-9622-ress-34-e20240330-suppl1-pt.pdf]

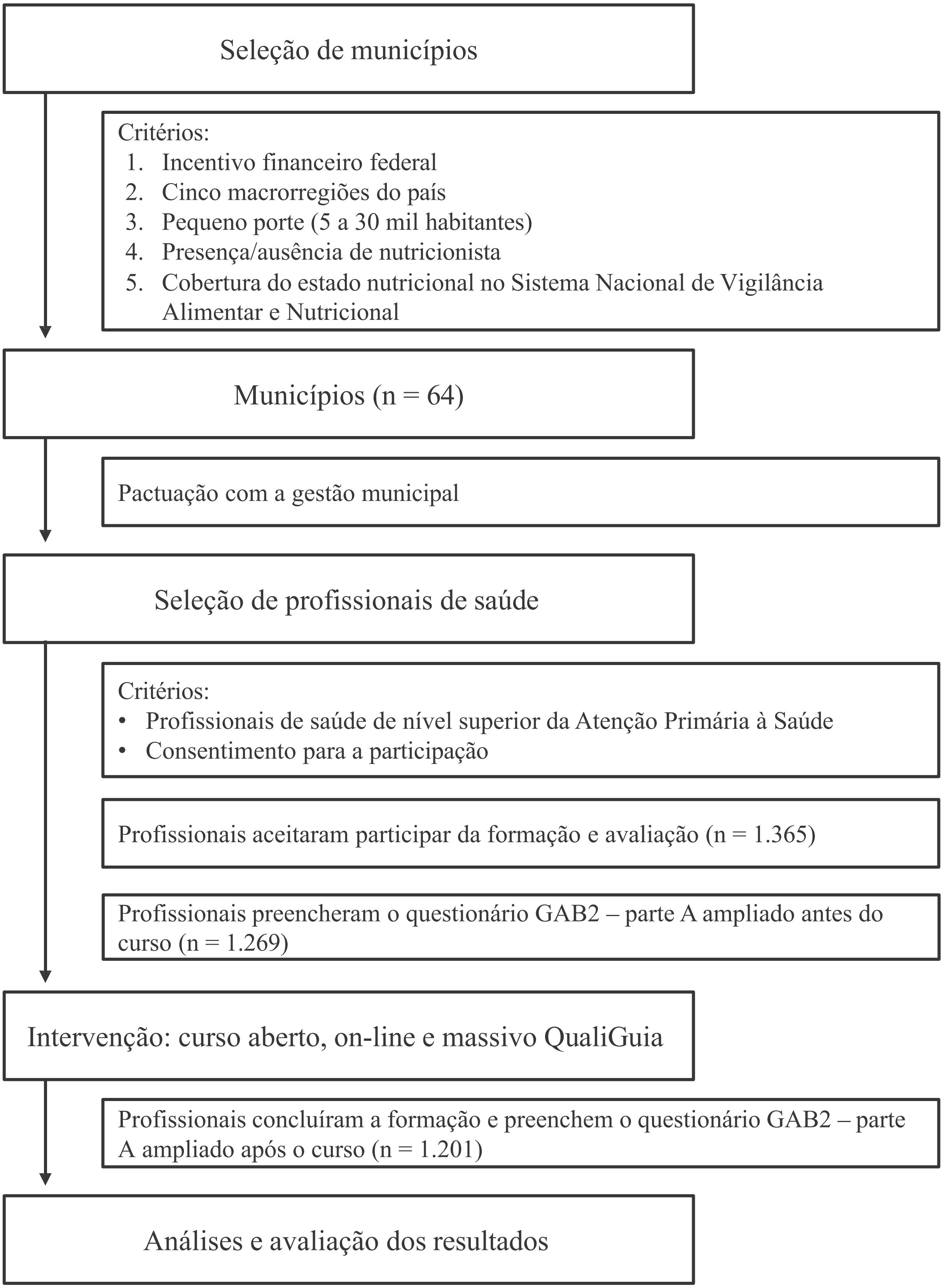
Seleção de municípios
Critérios:
1. Incentivo financeiro federal
2. Cinco macrorregiões do país
3. Pequeno porte (5 a 30 mil habitantes)
4. Presença/ausência de nutricionista
5. Cobertura do estado nutricional no Sistema Nacional de Vigilância Alimentar e Nutricional
Municípios (n = 64)
Pactuação com a gestão municipal
Seleção de profissionais de saúde
Critérios:
• Profissionais de saúde de nível superior da Atenção Primária à Saúde
• Consentimento para a participação
Profissionais aceitaram participar da formação e avaliação (n = 1.365)
Profissionais preencheram o questionário GAB2 – parte A ampliado antes do curso (n = 1.269)
Intervenção: curso aberto, on-line e massivo QualiGuia
Profissionais concluíram a formação e preenchem o questionário GAB2 – parte A ampliado após o curso (n = 1.201)
Análises e avaliação dos resultados

| Quadro suplementar n. 1 - Escala GAB 2 - parte A ampliada | |
|---|---|
| | Respostas |
| *No meu cotidiano de trabalho na Atenção Primária à Saúde:* | |
| 1) Eu consigo orientar os usuários do serviço de saúde a combinar alimentos na forma de refeições saudáveis. | Nada confiante (0)<br>Um pouco confiante (1)<br>Confiante (2)<br>Muito confiante (3) |
| 2) Eu consigo ensinar os usuários do serviço de saúde a analisar a lista de ingredientes dos rótulos de alimentos. | Nada confiante (0)<br>Um pouco confiante (1)<br>Confiante (2)<br>Muito confiante (3) |
| 3) Eu consigo estimular os usuários do serviço de saúde a realizar as refeições em família ou em companhia sempre que possível. | Nada confiante (0)<br>Um pouco confiante (1)<br>Confiante (2)<br>Muito confiante (3) |
| 4) Eu consigo estimular os usuários do serviço de saúde sobre os benefícios de comer com atenção. | Nada confiante (0)<br>Um pouco confiante (1)<br>Confiante (2)<br>Muito confiante (3) |
| 5) Eu consigo indicar fontes confiáveis de informação sobre alimentação saudável para os usuários do serviço de saúde. | Nada confiante (0)<br>Um pouco confiante (1)<br>Confiante (2)<br>Muito confiante (3) |
| 6) Eu consigo identificar no território os locais indicados para a aquisição de alimentos in natura ou minimamente processados. | Nada confiante (0)<br>Um pouco confiante (1)<br>Confiante (2)<br>Muito confiante (3) |
| 7) Eu consigo auxiliar os usuários do serviço de saúde a aprimorar suas habilidades culinárias. | Nada confiante (0)<br>Um pouco confiante (1)<br>Confiante (2)<br>Muito confiante (3) |
| 8) Eu consigo estimular o senso crítico dos usuários do serviço de saúde quanto à publicidade de alimentos. | Nada confiante (0)<br>Um pouco confiante (1)<br>Confiante (2)<br>Muito confiante (3) |
| 9) Eu consigo orientar os usuários do serviço de saúde sobre economizar com a compra de alimentos saudáveis. | Nada confiante (0)<br>Um pouco confiante (1)<br>Confiante (2)<br>Muito confiante (3) |
| 10) Eu consigo orientar os usuários do serviço de saúde como planejar o uso do tempo dedicado à alimentação. | Nada confiante (0)<br>Um pouco confiante (1)<br>Confiante (2)<br>Muito confiante (3) |
| 11) Eu conheço o conteúdo do Guia Alimentar para a População Brasileira, publicado pelo Ministério da Saúde. | Nada confiante (0)<br>Um pouco confiante (1)<br>Confiante (2)<br>Muito confiante (3) |
| 12) Eu consigo promover a alimentação saudável no meu território de atuação usando a abordagem do Guia Alimentar para a População Brasileira. | Nada confiante (0)<br>Um pouco confiante (1)<br>Confiante (2)<br>Muito confiante (3) |
| *Durante o atendimento individual:* | |
| 13) Eu consigo avaliar a alimentação dos usuários do serviço de saúde segundo os marcadores de consumo alimentar do SISVAN. | Nada confiante (0)<br>Um pouco confiante (1)<br>Confiante (2)<br>Muito confiante (3) |

| | |
|---|---|
| 14) Eu consigo estabelecer as prioridades nas orientações alimentares para os usuários do serviço de saúde. | Nada confiante (0)<br>Um pouco confiante (1)<br>Confiante (2)<br>Muito confiante (3) |
| 15) Eu consigo realizar orientações alimentares específicas considerando o ciclo da vida dos usuários do serviço de saúde. | Nada confiante (0)<br>Um pouco confiante (1)<br>Confiante (2)<br>Muito confiante (3) |
| 16) Eu consigo sugerir estratégias para os usuários do serviço de saúde reduzirem o consumo de alimentos e bebidas ultraprocessadas. | Nada confiante (0)<br>Um pouco confiante (1)<br>Confiante (2)<br>Muito confiante (3) |

**Tabela suplementar n. 1 - Comparação entre as características e o nível de conhecimento sobre o Guia Alimentar e os Protocolos de uso do Guia Alimentar dos profissionais de saúde que não concluíram a formação com aqueles que concluíram a formação (n= 1.269), Brasil, 2022-2024**

| | Profissionais que não concluíram o curso (n=68) | Profissionais que concluíram o curso (n=1.201) | p-valor |
|---|---|---|---|
| | n (%) | n (%) | |
| *Categorias profissionais* | | | |
| Enfermagem | 26 (38,2) | 404 (33,6) | |
| Odontologia | 14 (20,6) | 240 (20,0) | |
| Medicina | 16 (23,5) | 183 (15,2) | |
| Outras categorias profissionais | 12 (17,6) | 374 (31,1) | 0,069 |
| *Regiões do país* | | | |
| Nordeste | 29 (42,6) | 442 (36,8) | |
| Sudeste | 16 (23,5) | 381 (31,7) | |
| Norte | 15 (22,0) | 185 (15,4) | |
| Sul | 3 (4,4) | 155 (12,9) | |
| Centro-Oeste | 5 (7,3) | 38 (3,1) | 0,021 |
| *Raça/cor da pele* | | | |
| Branca | 35 (51,5) | 570 (47,5) | |
| Parda | 29 (42,6) | 517 (43,0) | |
| Preta | 2 (2,9) | 89 (7,4) | |
| Amarela | 2 (2,9) | 22 (1,8) | |
| Indígena | 0 (0,0) | 3 (0,2) | 0,510 |
| *Sexo* | | | |
| Feminino | 45 (66,2) | 953 (79,3) | |
| Masculino | 23 (33,8) | 248 (20,6) | 0,010 |
| *Idade (anos)* | | | |
| ≤ 29 | 16 (4,5) | 337 (95,5) | |
| 30-39 | 35 (6,7) | 489 (93,3) | |
| 40-49 | 15 (5,5) | 259 (94,5) | |
| ≥ 50 | 2 (1,8) | 110 (98,2) | 0,160 |
| *Tempo de atuação na profissão (anos)* | | | |
| ≤ 1 | 8 (11,8) | 203 (16,9) | |
| 2-3 | 21 (30,9) | 250 (20,8) | |
| 4-5 | 13 (19,1) | 158 (13,2) | |
| ≥ 6 | 26 (38,2) | 590 (49,1) | 0,062 |
| *Conhecimento sobre o Guia Alimentar* | | | |
| Sim | 38 (55,9) | 636 (53,0) | |
| Não | 30 (44,1) | 565 (47,0) | 0,638 |

| | Profissionais que não concluíram o curso (n=68) | Profissionais que concluíram o curso (n=1.201) | p-valor |
|---|---|---|---|
| | n (%) | n (%) | |
| *Entre aqueles que conhecem o Guia Alimentar para a População Brasileira:* | | | |
| Ouviu falar: | | | |
| Sim | 18 (26,5) | 326 (27,1) | |
| Não | 50 (73,5) | 875 (72,9) | 0,903 |
| Participou de formação: | | | |
| Sim | 11 (16,18) | 186 (15,5) | |
| Não | 57 (83,8) | 1.015 (84,5) | 0,879 |
| Utiliza na prática clínica: | | | |
| Sim | 10 (14,7) | 142 (11,8) | |
| Não | 58 (85,3) | 1.059 (88,2) | 0,476 |
| *Conhecimento sobre os Protocolos de uso do Guia Alimentar:* | | | |
| Sim | 26 (38,2) | 495 (41,2) | |
| Não | 42 (61,8) | 706 (58,8) | 0,627 |
| *Entre aqueles que conhecem os Protocolos de uso do Guia Alimentar:* | | | |
| Ouviu falar: | | | |
| Sim | 10 (14,7) | 237 (19,7) | |
| Não | 58 (85,3) | 964 (80,3) | 0,308 |
| Participou de formação: | | | |
| Sim | 9 (13,2) | 179 (14,9) | |
| Não | 59 (86,8) | 1.022 (85,1) | 0,706 |
| Utiliza na prática clínica: | | | |
| Sim | 8 (11,8) | 87 (7,2) | |
| Não | 60 (88,2) | 1.114 (92,8) | 0,168 |

**Tabela suplementar n. 2 - Comparação entre a percepção de autoeficácia (média, desvio padrão, IC95%) antes da intervenção dos profissionais de saúde que não concluíram a formação com aqueles que concluíram a formação (n= 1.269), Brasil, 2022-2024**

| | Profissionais que não concluíram o curso (n=68) | Profissionais que concluíram o curso (n=1.201) | p-valor |
|---|---|---|---|
| *Escore de autoeficácia pré-intervenção:* | | | |
| Média | 23,1 | 23,7 | |
| Desvio padrão | 10,7 | 10,7 | |
| IC 95% | 20,5-25,7 | 23,1-24,3 | 0,671 |
