# Supplementary material for: Change in healthcare professionals’ perception of self-efficacy for providing dietary advice after completing a massive open online course: an uncontrolled community trial in 64 Brazilian municipalities, 2022-2024
Source: Epidemiol Serv Saude. 2025 Jun 13;34:e20240330. doi: 10.1590/S2237-96222025v34e20240330.en (PMC12176444; doi:10.1590/S2237-96222025v34e20240330.en)
Supplement: Translation

# Translation: Mudança na percepção de autoeficácia de profissionais de saúde para realizar orientações alimentares após a participação em um curso aberto, on-line e massivo: ensaio comunitário não controlado em 64 municípios brasileiros, 2022-2024

**DOI:** 10.1590/S2237-96222025v34e20240330.pt

**Authors:** Vanessa Del Castillo Silva Couto, Adhara Brandão Lima Vanhoz, Kamila Tiemann Gabe, Cleyton Zanardo de Oliveira, Patrícia Constante Jaime

**Affiliations:** 1 Universidade de São Paulo, Núcleo de Pesquisas Epidemiológicas em Nutrição e Saúde, São Paulo, SP, Brasil; 2 Escritório do Programa de Apoio ao Desenvolvimento Institucional do Sistema Único de Saúde, BP – A Beneficência Portuguesa de São Paulo, São Paulo, SP, Brasil

**Keywords:** Guias Alimentares, Política Nutricional, Educação Profissional em Saúde Pública, Educação a Distância, Estudo de Avaliação

## Abstract

**Objetivo:**

Avaliar a mudança na percepção de autoeficácia de profissionais de saúde da Atenção Primária à Saúde para realizar orientações alimentares utilizando os Protocolos de uso do *Guia Alimentar para a População Brasileira* após a realização de um curso aberto, on-line e massivo (MOOC, sigla em inglês).

**Métodos:**

Ensaio comunitário não controlado. Profissionais de saúde de nível superior da Atenção Primária à Saúde de 64 municípios de pequeno porte no Brasil formam a população do estudo. A intervenção foi um curso on-line, aberto e massivo. O desfecho foi medido por uma escala validada que mensura a percepção de autoeficácia para realizar orientações alimentares com base nos Protocolos de uso do Guia Alimentar. O escore médio foi calculado antes e após a intervenção e a mudança do escore foi testada com teste t pareado (p-valor<0,05).

**Resultados:**

Entre os 1.201 profissionais de saúde, a média do escore de autoeficácia passou de 23,71 para 37,31 após a intervenção (p-valor<0,001), aumento que foi significativo em todas as categorias profissionais.

**Conclusão:**

O curso on-line, aberto e massivo aumentou a percepção de autoeficácia dos profissionais de saúde da Atenção Primária à Saúde para realizar orientações alimentares individuais baseadas nos Protocolos de uso do Guia Alimentar para a População Brasileira.

**Aspectos éticos**

A presente pesquisa respeitou os princípios éticos, obtendo os seguintes dados de aprovação: :

Comitê de ética em pesquisa: Número do parecer, Data de aprovação, Certificado de apresentação de apreciação ética

Hospital Beneficência Portuguesa de São Paulo: 6.121.289, 29/8/2022, 60875322.0.1001.5483

Universidade de São Paul: 5.719.038, 24/10/2022, 60875322.0.2001.5421

Registro do consentimento livre e esclarecido: Obtido de todos os participantes antes da coleta.

## Introdução

O *Guia Alimentar para a População Brasileira* (doravante referido como Guia Alimentar) é o documento que estabelece recomendações de alimentação saudável no Brasil (1) baseado em evidências que reforçam sua importância e atualidade (2). Pioneiro ao incorporar a classificação Nova (3), que considera o nível e o propósito do processamento de alimentos, o Guia Alimentar aborda também padrões alimentares, comensalidade, cultura alimentar e elenca estratégias para superar barreiras para uma alimentação saudável (4). O Guia Alimentar tem sido implementado no Sistema Único de Saúde (SUS) e na Atenção Primária à Saúde (APS) (5), servindo como referência para o desenvolvimento de outras ferramentas de apoio para o cuidado nutricional e a prática clínica, como os Protocolos de uso do *Guia Alimentar para a População Brasileira* na orientação alimentar individual (doravante chamados Protocolos) (6–8). Os Protocolos, divididos em cinco fascículos organizados por fases do curso da vida (9–13), apoiam os profissionais de saúde na orientação alimentar individual, por meio de um processo sistematizado em três etapas: diagnóstico do consumo alimentar (14,15), organização das recomendações prioritárias com base em um fluxograma decisório, e realização de orientações alimentares específicas (6). 

A qualificação profissional é uma estratégia de médio prazo para a implementação de Guias Alimentares e é uma responsabilidade prevista no SUS (5), que tem o dever de assegurar a qualificação dos profissionais de saúde para ofertar o melhor cuidado (16). A Política Nacional de Educação Permanente em Saúde orienta que a formação em saúde seja baseada nas necessidades de saúde identificadas, utilizando atividades reflexivas que consideram os desafios do processo de trabalho, envolvendo diversos atores, com o objetivo de promover transformações nas práticas de cuidado (17). 

Os cursos on-line massivos e abertos (tradução livre do termo *Massive Open Online Course*, MOOC) são uma modalidade de formação caracterizada pela disseminação de conteúdos educacionais de forma ampla, por meio da internet, para grandes grupos de educandos distribuídos em territórios extensos. Os MOOC têm sido amplamente utilizados em formações na área da saúde ao redor do mundo (18) e no contexto do SUS, como ferramenta de qualificação de profissionais de saúde (19–21).

Com base neste cenário, um MOOC sobre os Protocolos foi desenvolvido e validado com o objetivo de qualificar profissionais da APS. Este estudo teve como objetivo avaliar a mudança na percepção da autoeficácia de profissionais da Atenção Primária à Saúde para realizar orientações alimentares utilizando os Protocolos de uso do Guia Alimentar após a realização do curso.

## Métodos

### Delineamento e contexto do estudo

Este estudo foi um ensaio comunitário não controlado. Os participantes foram profissionais de saúde de nível superior da APS de 64 municípios de pequeno porte do Brasil. A intervenção constituiu na qualificação profissional com um MOOC, denominado QualiGuia. O curso teve como objetivo apoiar a incorporação dos Protocolos na prática clínica dos profissionais de saúde de APS. Esperava-se que o curso influenciasse na mudança na percepção de autoeficácia para realizar orientações alimentares utilizando os Protocolos. O desfecho de interesse foi aferido antes e depois da intervenção, utilizando um questionário validado para mensurar a percepção de autoeficácia. Este estudo foi desenvolvido entre 2022 e 2023, como parte de um projeto, também denominado QualiGuia, do Programa de Apoio ao Desenvolvimento Institucional do SUS (PROADI-SUS) conduzido pela BP - A Beneficência Portuguesa de São Paulo (22). 

### Participantes do estudo

Foram selecionados 64 municípios de pequeno porte (5 a 30 mil habitantes), sendo 62 da lista principal e 2 suplentes, que aceitaram participar e oferecer o MOOC aos seus profissionais de saúde. A seleção seguiu os seguintes critérios: receber financiamento federal para desenvolvimento de ações de Alimentação e Nutrição, distribuição nas cinco macrorregiões do país, representação de municípios com e sem nutricionista na APS e bons desempenhos na cobertura do estado nutricional no Sistema Nacional de Vigilância Alimentar e Nutricional. O MOOC foi ofertado em cada município e 1.365 profissionais de saúde da APS iniciaram o MOOC. Destes, 1.269 preencheram o questionário GAB2 – parte A ampliado antes da formação e 1.201 concluíram o curso e preencheram o questionário novamente, após a formação. 

### Desenho da intervenção: MOOC QualiGuia

O MOOC foi desenvolvido e validado no âmbito do projeto QualiGuia do PROADI-SUS. Este curso foi desenvolvido com base na necessidade identificada pelo Ministério da Saúde de implementar os Protocolos, lançados entre 2021 e 2022, para apoiar a orientação alimentar individual por fases do curso da vida e avançar na implementação das recomendações do Guia Alimentar na APS. 

Classificado como um MOOC por ser on-line, acessível a um público amplo e abrangendo um grande território, o curso explora diferentes ferramentas pedagógicas de ensino à distância. O MOOC QualiGuia está disponível na plataforma da Universidade Aberta do SUS (23).

O curso teve como referencial teórico a) A Política Nacional de Educação Permanente em Saúde, que orienta a formação baseada em necessidades identificadas no cotidiano para promover reflexões e mudanças no cuidado (17); b) A Política Nacional de Atenção Básica, reforçando a APS como o lócus para a qualificação do cuidado nutricional (24); e c) A Política Nacional de Alimentação e Nutrição, que define diretrizes para a atenção nutricional e promoção da alimentação adequada e saudável (25). 

O conteúdo central do curso é baseado no Guia Alimentar e nos Protocolos. Estudos de caso baseados no contexto da APS, apresentados como vídeos animados, são o principal recurso de aprendizagem para estimular a reflexão sobre as formas de utilização dos Protocolos como ferramenta para o cuidado nutricional. 

O curso foi validado por um painel com 15 especialistas que avaliaram clareza, relevância e adequação ao referencial teórico (26). As avaliações escritas foram analisadas por meio do Índice de Validade de Conteúdo (IVC). Adicionalmente, dois grupos focais realizados foram analisados pela abordagem compreensiva com análise temática de conteúdo (26,27). O IVC foi calculado pela proporção de avaliações 3 e 4 na escala Likert, com todas as atividades atingindo IVC>0,8 (ponto de corte), validando o curso. A análise temática identificou três categorias: 1) Pertinência da proposta e reconhecimento para a APS:​ destacando o papel estratégico do MOOC na implementação do Guia Alimentar. 2) Adequação do conteúdo: reforçando a qualidade do material e da metodologia de ensino-aprendizagem. 3) Desafios para a implementação do curso na APS: apontando reflexões relevantes para a implementação. O formato e estrutura do curso não sofreram modificações e as sugestões do painel de especialistas foram consideradas e incorporadas na versão final.

O curso é autoinstrucional, tem carga horária de 30 horas e é direcionado para profissionais de saúde de nível superior que realizam atendimentos individuais. 

Neste estudo, os participantes tiveram um mês para inscrição e acesso inicial, cinco meses para completar a formação e um mês adicional para emitir o certificado e salvar os materiais.

### Fonte e coleta de dados

Os dados foram coletados entre janeiro e novembro de 2023 com o *Research Electronic Data Capture* - REDCap (28). Os profissionais foram convidados a preencher a escala GAB 2 – parte A ampliada on-line, imediatamente antes do início e após a conclusão do MOOC. Também foram coletadas informações sociodemográficas e de conhecimento prévio sobre o Guia Alimentar e os Protocolos no momento pré-curso. 

### Desfecho de interesse e variáveis do estudo

A percepção de autoeficácia reflete a confiança ou competência pessoal em determinada habilidade para realizar um comportamento específico (29). A autoeficácia pode indicar como as pessoas agirão, contribuindo para determinar o que podem fazer com as habilidades e conhecimentos adquiridos no futuro (30). Ademais, a crença na autoeficácia influencia as escolhas e decisões, uma vez que as pessoas tendem a preferir realizar atividades nas quais se sintam confiantes e competentes. 

A “escala de mensuração da autoeficácia de profissionais de saúde da Atenção Básica para aplicar o Guia Alimentar” (nomeada GAB 2 – parte A) é uma escala autoaplicável de 12 itens em formato Likert de quatro pontos (pontuação de 0 a 3), possibilitando pontuação de 0 a 36 pontos (31). Quatro itens adicionais foram desenvolvidos para captar as dimensões: 1) diagnóstico da alimentação segundo os marcadores de consumo alimentar. 2) ordenação de prioridades segundo o fluxograma decisório. 3) diferenciação de orientações específicas por fase do curso da vida. 4) um item síntese das estratégias de superação de obstáculos, com a regra de ouro do Guia Alimentar. 

Os itens adicionais foram elaborados e discutidos com pesquisadoras com experiência no desenvolvimento de escalas, no Guia Alimentar e nos Protocolos. Após revisão, uma segunda versão dos itens foi avaliada por uma nutricionista da APS para verificar a extensão da compreensão dos itens. 

Os itens foram definidos e a escala ampliada para 16 itens em formato Likert de quatro pontos (pontuação de 0 a 3), totalizando de 0 a 48 pontos. Nomeada GAB 2 – parte A ampliada, sua consistência interna foi avaliada pelo Alfa de Cronbach e uma Análise Fatorial foi realizada para verificar o agrupamento dos itens. A avaliação utilizou dados de antes da intervenção de uma subamostra de 1.039 participantes coletados em julho de 2023. 

A compreensão das questões foi satisfatória, a nova escala apresentou média: 23,6; desvio padrão: 10,7 e alfa de Cronbach de 0,955 (IC95% 0,951; 0,959). A escala de 12 itens apresentou média: 17,8; desvio padrão: 8,6 e alfa de Cronbach de 0,942 (IC95% 0,970; 0,976). A análise fatorial apresentou 2 componentes. O componente 1: estratégias para superação de obstáculos para uma alimentação saudável, reúne itens relacionados a confiança em apoiar os usuários na adoção de uma alimentação saudável segundo as recomendações do Guia Alimentar. O componente 2: orientação alimentar individual baseada no Guia Alimentar, reúne itens que consolidam o conhecimento das recomendações do Guia Alimentar e as aplicações práticas ao cuidado segundo o Guia Alimentar e os Protocolos.

A adaptação da escala foi válida para mensurar o desfecho de interesse, a percepção de autoeficácia de profissionais de saúde da APS para realizar orientações alimentares individuais baseadas nos Protocolos e nas recomendações do Guia Alimentar (Quadro suplementar 1).

Adicionalmente, foram produzidas variáveis com as informações sociodemográficas (categoria profissional, macrorregião, data de nascimento, sexo, raça/cor da pele e tempo de atuação na profissão) e sobre o conhecimento prévio do Guia Alimentar e dos Protocolos, com respostas iniciais de “sim” e “não”. Para aqueles que responderam “sim”, foram oferecidas opções adicionais: “só ouviu falar”, “já participou de alguma formação” e “utiliza na prática clínica”.

### Análise de dados e mensuração

Os dados foram analisados no software Stata SE 16 e R Studio (Versão 4.3.2). A caracterização dos participantes foi feita com análise descritiva das variáveis qualitativas descritas em frequência. Para aferir a mudança na percepção de autoeficácia, foi calculado o escore total (média) da escala GAB 2 – parte A ampliada para cada indivíduo, comparando os escores de antes e depois da realização do curso com o teste t de Student pareado. Associações entre características sociodemográficas, conhecimento prévio sobre o Guia Alimentar e os Protocolos e o aumento da autoeficácia (considerando o delta da variação do escore) foram analisadas utilizando os testes t de Student e Anova. Análises post-hoc de Tukey identificaram quais categorias apresentaram associação significativa na etapa anterior. Adicionalmente, comparou-se as características dos profissionais que desistiram do MOOC com aqueles que concluíram o curso, aplicando os testes Exato de Fisher, Qui Quadrado e t de Student para verificar a similaridade entre os grupos. A significância estatística foi aceita com p-valor<0,050.

A Figura suplementar 1 apresenta um fluxograma das etapas metodológicas do estudo.

## Resultados

Entre os 1.201 profissionais de saúde, a maioria era do sexo feminino (79,3%), com 50,5% se autodeclarando pessoas negras e 47,5% brancas. A faixa etária mais prevalente foi de 30 a 39 anos (40,9%). A categoria profissional mais representada foi a Enfermagem (33,6%), seguida por Odontologia (20,0%) e Medicina (15,2%). Cerca de 49,1% tinham mais de seis anos de atuação na profissão. As regiões com maior participação foram Nordeste (36,8%) e Sudeste (31,7%) (Tabela 1). Ademais, 53,0% dos profissionais conheciam o Guia Alimentar, sendo que, entre estes, 51,3% ouviram falar, enquanto 22,4% utilizavam na prática clínica. Em relação aos Protocolos, 41,2% afirmaram conhecê-los e destes, 48,0% ouviram falar e 17,6% utilizavam na prática clínica (Tabela 2). 

**Tabela 1:** Características dos profissionais de saúde. Brasil, 2022-2024 (n=1.201)

Variáveis		n (%)
**Categorias profissionais**		
	Enfermagem	404 (33,6)
	Odontologia	240 (20,0)
	Medicina	183 (15,2)
	Fisioterapia	100 (8,3)
	Nutrição	82 (6,8)
	Psicologia	70 (5,8)
	Educação Física	42 (3,5)
	Serviço Social	40 (3,3)
	Farmácia, Fonoaudiologia e Terapia Ocupacional	40 (3,3)
**Macrorregião do país**		
	Nordeste	442 (36,8)
	Sudeste	381 (31,7)
	Norte	185 (15,4)
	Sul	155 (12,9)
	Centro-Oeste	38 (3,2)
**Raça/cor da pele**		
	Branca	570 (47,5)
	Parda	517 (43,0)
	Preta	89 (7,4)
	Amarela	22 (1,8)
	Indígena	3(0,2)
Sexo		
	Feminino	953 (79,3)
	Masculino	248 (20,6)
**Idade** (**anos) (6 dados faltantes**)		
	≤29	350 (29,1)
	30-39	491 (40,9)
	40-49	248 (20,6)
	≥50	106 (8,8)
**Tempo de atuação na profissão** (anos)		
	≤1	203 (16,9)
	2-3	250 (20,8)
	4-5	158 (13,2)
	≥6	590 (49,1)

**Tabela 2:** Conhecimento prévio dos profissionais de saúde sobre o *Guia Alimentar para a População Brasileira* e os Protocolos de uso. Brasil, 2022-2024

Conhecimentos prévios		n (%)
**Conhecimento sobre o Guia Alimentar** (n=1.201)		
	Conhece o *Guia Alimentar para a População Brasileira*	636 (53,0)
**Entre aqueles que conhecem o Guia Alimentar** (n=635)		
	Ouviu falar	326 (51,3)
	Participou de formação	167 (26,3)
	Utiliza na prática clínica	123 (19,4)
	Participou de formação e utiliza na prática clínica	19 (3,0)
**Conhecimento sobre os Protocolos de uso do Guia Alimentar** (n=1.201)		
	Conhece os Protocolos de uso do Guia Alimentar	495 (41,2)
**Entre aqueles que conhecem os Protocolos de uso do Guia Alimentar** (n=494)		
	Ouviu falar	237 (48,0)
	Participou de formação	170 (34,4)
	Utiliza na prática clínica	78 (15,8)
	Participou de formação e utiliza na prática clínica	9 (1,8)

A Figura 1 apresenta a distribuição do escore de percepção de autoeficácia dos profissionais após a intervenção. Antes da intervenção, o escore médio de autoeficácia era de 23,7 (mediana=24; desvio padrão=10,^7^). Após a intervenção, o escore médio aumentou para 37,3 (mediana=36; desvio padrão=8,^9^), com diferença estatisticamente significativa (p-valor<0,001). 

**Figura 1:** Distribuição do escore de percepção de autoeficácia para realizar orientações alimentares com base nos Protocolos de uso do *Guia Alimentar para a População Brasileira* antes e após a intervenção. Brasil, 2022-2024 (n=1.201)

Na Tabela 3 são apresentados os resultados por categoria profissional, indicando aumento significativo na percepção de autoeficácia em todas as categorias profissionais após a intervenção (p-valor<0,001). Profissionais da Nutrição e Educação Física apresentaram as menores variações percentuais, enquanto as demais categorias profissionais mostraram aumento superior de 50%. 

**Tabela 3:** Diferença no escore de percepção de autoeficácia (média, desvio padrão, IC95%) antes e após a intervenção, por categoria profissional. Brasil, 2022–2024 (n=1.201)

			Escore de autoeficácia pré-intervenção			Escore de autoeficácia pós-intervenção			Δ			p-valor
Categorias profissionais		n	Média	Desvio padrão	IC95%	Média	Desvio padrão	IC95%	Diferença das médias	Delta Percentual	IC95%	
	Enfermagem	404	24,3	9,4	23,4; 25,2	37,6	8,2	36,8; 38,4	13,3	54,9	14,3; 12,3	<0,001
	Odontologia	240	20,0	10,4	18,7; 21,3	35,8	10,1	34,6; 37,1	15,8	79,2	17,3; 14,4	<0,001
	Medicina	183	24,8	8,9	23,5; 26,1	39,3	7,5	38,2; 40,4	14,4	58,1	15,8; 13,1	<0,001
	Fisioterapia	100	21,9	10,3	19,9; 24,0	37,9	8,8	36,1; 39,6	16,0	72,8	18,3; 13,7	<0,001
	Nutrição	82	38,2	8,6	36,3; 40,0	43,1	6,2	41,7; 44,5	4,9	12,9	6,3; 3,5	<0,001
	Psicologia	70	19,2	10,9	16,6; 21,8	32,9	9,7	30,6; 35,3	13,7	71,3	16,8; 10,7	<0,001
	Educação Física	42	25,9	8,8	23,1; 28,6	33,8	9,1	31,0; 36,7	7,9	30,7	10,8; 5,1	<0,001
	Serviço Social	40	21,3	11,9	17,5; 25,1	34,4	11,2	30,9; 38,0	13,1	61,4	16,5; 9,5	<0,001
	Farmácia, Fonoaudiologia e Terapia Ocupacional	40	17,8	10,0	14,6; 21,1	34,8	6,9	32,6; 37,0	16,9	94,8	20,4; 13,4	<0,001

A Tabela 4 detalha as associações entre características sociodemográficas, de conhecimento prévio sobre o Guia Alimentar e os Protocolos e o aumento da autoeficácia. Entre as características sociodemográficas, raça/cor da pele, profissão e macrorregião foram significativamente associadas ao aumento da autoeficácia (p-valor<0,050). A análise revelou que a associação foi maior entre indivíduos brancos e pretos. Profissionais de Nutrição e Educação Física apresentaram menor aumento da autoeficácia em comparação às demais categorias profissionais como Enfermagem, Odontologia, Medicina, Fisioterapia, Serviço Social, Psicologia e Farmácia, Fonoaudiologia e Terapia Ocupacional. Para a variável macrorregião, a associação foi maior entre as macrorregiões Sul e Sudeste. 

**Tabela 4:** Associação entre características sociodemográficas e conhecimento prévio sobre o *Guia Alimentar para a População Brasileira* e Protocolos de uso com o aumento na autoeficácia após o curso (delta da variação dos escores). Brasil, 2022–2024

Variáveis	n	Média	Desvio padrão	p-valor
**Categoria profissional**				<0,001
Enfermagem	404	13,3	10,1	
Odontologia	240	15,8	11,5	
Medicina	183	14,4	9,2	
Fisioterapia	100	16,0	11,6	
Nutrição	82	4,9	6,5	
Psicologia	70	13,7	12,8	
Educação Física	42	8,0	9,1	
Serviço Social	40	13,1	11,1	
Farmácia, Fonoaudiologia e Terapia Ocupacional	40	16,9	10,9	
**Macrorregião do país**				0,043
Nordeste	442	13,5	10,8	
Sudeste	381	12,9	10,9	
Norte	185	13,3	10,6	
Sul	155	16,0	10,6	
Centro Oeste	38	12,4	9,3	
**Raça/cor da pele**				0,006
Branca	570	14,6	10,8	
Parda	517	13,0	10,7	
Preta	89	11,3	10,7	
Amarela/Indígena	25	11,2	9,3	
Sexo				0,261
Feminino	953	13,8	10,9	
Masculino	248	12,9	10,1	
**Idade** (**anos) (6 dados faltantes**)				0,149
≤29	350	14,3	11,58	
30-39	491	13,51	10,18	
40-49	248	13,24	10,42	
≥50	106	11,64	10,05	
**Tempo de atuação na profissão** (anos)				0,13
≤1	203	13,0	9,8	
2-3	250	13,8	11,1	
4-5	158	13,1	11,6	
≥6	590	13,8	10,6	
**Conhecimento prévio sobre o Guia Alimentar**				
Conhece o Guia Alimentar	636	11,7	10,1	<0,001
**Entre aqueles que conhecem o Guia Alimentar**				
Ouviu falar	326	13,1	9,8	0,336
Participou de formação	186	11,8	10,6	0,011
Utiliza na prática clínica	142	7,9	8,7	<0,001
**Conhecimento sobre os Protocolos de uso do Guia Alimentar**				
Conhece os Protocolos de uso do Guia Alimentar	495	11,1	10,1	<0,001
**Entre aqueles que conhecem os Protocolos de uso do Guia Alimentar**				
Ouviu falar	237	12,4	10,3	0,061
Participou de formação	179	11,2	10,0	0,001
Utiliza na prática clínica	87	7,1	8,7	<0,001

O conhecimento prévio sobre o Guia Alimentar e os Protocolos também foi associado ao aumento da autoeficácia (p-valor<0,050). Em ambos os casos, o aumento foi significativo entre aqueles que já haviam participado de formações ou que utilizavam em sua prática clínica (Tabela 4). 

Por fim, ao comparar os grupos que concluíram e não concluíram o curso, diferenças significativas foram observadas em relação à macrorregião e ao sexo. No entanto, ambos os grupos foram estatisticamente semelhantes quanto ao conhecimento prévio sobre o Guia Alimentar e os Protocolos (Tabela suplementar 1). Além disso, as médias dos escores de autoeficácia antes da intervenção também não apresentaram diferenças significativas entre os grupos (Tabela suplementar 2).

## Discussão

Os resultados deste estudo corroboram a produção de evidências sobre o papel da qualificação profissional no aumento da autoeficácia para realizar orientações alimentares baseadas nos Protocolos e no Guia Alimentar. Após a intervenção, observou-se um aumento significativo da percepção de autoeficácia tanto para o conjunto dos profissionais quanto para cada categoria profissional analisada. 

A principal limitação deste estudo é a ausência de grupo controle, o que restringe a capacidade de atribuir as mudanças observadas na autoeficácia exclusivamente à formação com o MOOC, pois os resultados podem ter sido influenciados por fatores externos não mensurados. Outra possível limitação é o viés de desejabilidade social, uma vez que os participantes podem ter respondido à escala de autoeficácia de forma mais positiva ao concluírem o MOOC, acreditando que esse seria o comportamento esperado, o que nem sempre reflete a real percepção de autoeficácia. No entanto, é importante destacar que a autoeficácia só pode ser aferida por meio de escalas autoaplicáveis. Por outro lado, o estudo apresentou fortalezas, como sua ampla capilaridade, com participação expressiva de profissionais de todas as macrorregiões e em um contexto similar à realidade das formações realizadas nessa modalidade. Assim, o estudo se apresenta como uma evidência da efetividade deste MOOC. 

A autoeficácia é um constructo que pode contribuir para a avaliação de implementação, uma vez que mensura a confiança em aplicar um conhecimento adquirido na prática profissional (31). Estudos anteriores indicaram que há maior relação entre a elevada autoeficácia e mudanças nas práticas do que entre conhecimento elevado e mudanças nas práticas (32). Portanto, o aumento significativo da autoeficácia para realizar orientações alimentares após a formação com o MOOC aponta seu potencial em apoiar mudanças nas práticas de cuidado dos profissionais de saúde, contribuindo para a implementação dos Protocolos e do Guia Alimentar na APS.

O MOOC favoreceu o aumento da autoeficácia em todas as categorias profissionais. A maioria das categorias profissionais apresentou um aumento relativo de mais de 50% na percepção de autoeficácia após a intervenção. As diferenças nas médias de autoeficácia entre as categorias profissionais diminuíram após a intervenção, sinalizando um nivelamento entre elas. 

A categoria Nutrição apresentou os escores mais elevados em ambos os momentos, mas o menor aumento relativo na autoeficácia. Essa categoria centraliza o núcleo de saber em Alimentação e Nutrição, mas ainda assim se beneficiou da formação, provavelmente devido à característica dos Protocolos de apoiar a sistematização do cuidado individual na prática clínica (33). 

Esses achados ajudam a compreender que o Guia Alimentar e os Protocolos não se restringem ao núcleo de atuação da Nutrição, mas abordam a alimentação no campo da saúde coletiva, com ênfase no propósito e processamento dos alimentos, em padrões alimentares, hábitos, ambiente alimentar. Além de trazer um novo paradigma para a alimentação e nutrição, conseguem endereçar a principal recomendação na regra de ouro e tem uma série de estratégias para superar os obstáculos para uma alimentação saudável (34). Esses documentos contribuem para borrar os limites entre os saberes específicos da Nutrição e os saberes interdisciplinares que compõem o campo da saúde coletiva. O MOOC atuou como mediador desses saberes, promovendo ganhos de confiança tanto entre categorias específicas quanto no conjunto das práticas interprofissionais. 

A alimentação e nutrição são temas recorrentes no cuidado da APS, dada a sua relação com a má nutrição e doenças crônicas. Por isso, são temas frequentemente abordados nos espaços de cuidado por diferentes profissionais de saúde. O aumento da autoeficácia para realizar orientações alimentares baseadas no Guia Alimentar são um resultado positivo que reflete o interesse e o compromisso dos profissionais em aprimorar suas práticas para o cuidado nutricional, além de sinalizar avanços na prática de colaboração interprofissional (35). Esse resultado reforça a necessidade identificada de formações específicas para a orientação alimentar individual, permitindo que os profissionais se sintam mais confiantes em realizar essa prática de cuidado.

O conhecimento prévio sobre o Guia Alimentar e os Protocolos esteve associado ao aumento da autoeficácia entre aqueles que já participaram de formações anteriores e/ou já utilizavam esses materiais na prática clínica. Por outro lado, não houve associação entre o aumento da autoeficácia e somente ter ouvido falar dos documentos. Esse achado reforça a relação entre conhecimento e autoeficácia. Ainda assim, reconhece-se que essa modalidade de curso se fundamenta na ideia de que a formação ocorre ao longo da trajetória profissional, pela interação entre o conhecimento prévio, o cotidiano de trabalho e os novos conhecimentos necessários para qualificar as práticas de cuidado (36). 

Os MOOC podem ser ferramentas estratégicas para a formação de profissionais de saúde no SUS (18,37). Este estudo aponta que o MOOC é capaz de ampliar a confiança dos profissionais de saúde para realizar orientações alimentares baseadas nos Protocolos, independente de seu nível de conhecimento prévio, promovendo um nivelamento da autoeficácia entre as categorias profissionais. 

A análise também revelou que os profissionais que não concluíram o MOOC eram estatisticamente semelhantes aos que concluíram em termos de conhecimento prévio sobre o Guia Alimentar, os Protocolos e os escores de autoeficácia antes da intervenção. Entretanto, diferenças significativas foram encontradas quanto à macrorregião, o que reforça as disparidades regionais na execução da APS no Brasil (38). Esse achado destaca o desafio de considerar os contextos locais ao planejar ações de formação em saúde.

Apesar dos avanços, permanece o desafio de planejar e executar qualificações profissionais capilarizadas no SUS, garantindo que as melhores práticas de cuidado sejam ofertadas à população (17). Entre os desafios das formações profissionais baseadas em MOOC, destaca-se a necessidade de assegurar que sejam reflexivas e conectadas ao cotidiano de trabalho, além de objetivar a promoção de mudanças nas práticas de cuidado nos serviços de saúde (39). 

Futuros estudos deverão avaliar o impacto desta intervenção em diferentes níveis, considerando desde as reações dos profissionais ao MOOC até mudanças no comportamento, em indicadores e outros efeitos no sistema de saúde local. Desta forma, será possível compreender melhor as múltiplas dimensões que compõem a avaliação de impacto deste MOOC, levando em conta os desafios da APS e o contexto local (40). 

Em conclusão, o curso MOOC contribuiu para o aumento da percepção de autoeficácia dos profissionais de saúde na realização de orientações alimentares individuais baseadas nos Protocolos de uso do Guia Alimentar. Esse achado reforça a evidência de que um MOOC ofertado pelo SUS pode qualificar profissionais de saúde e assegurar a oferta de formações de qualidade no sistema de saúde.

## Data Availability

O banco de dados utilizados na pesquisa não está disponível publicamente para proteger a privacidade dos participantes do estudo. Entretanto, um banco anonimizado pode ser disponibilizado pelo autor principal mediante solicitação.
